# Induction of DNA damage by deguelin is mediated through reducing DNA repair genes in human non-small cell lung cancer NCI-H460 cells

**DOI:** 10.3892/or.2012.1622

**Published:** 2012-01-04

**Authors:** BIN-CHUAN JI, CHIEN-CHIH YU, SU-TSO YANG, TE-CHUN HSIA, JAI-SING YANG, KUANG-CHI LAI, YANG-CHING KO, JEN-JYH LIN, TUNG-YUAN LAI, JING-GUNG CHUNG

**Affiliations:** 1 Division of Chest Medicine, Department of Internal Medicine, Changhua Christian Hospital, Changhua 500; 2 School of Pharmacy, China Medical University, Taichung 404; 3 School of Chinese Medicine, China Medical University, Taichung 404; 4 Department of Radiology, China Medical University Hospital, Taichung 404; 5 Department of Internal Medicine, China Medical University Hospital, Taichung 404; 6 Department of Pharmacology, China Medical University, Taichung 404; 7 Department of Surgery, China Medical University Beigang Hospital, Yunlin 651; 8 School of Medicine, China Medical University, Taichung 404; 9 Division of Pulmonary and Critical Care Medicine, Department of Internal Medicine, St. Martin De Porres Hospital, Chiayi 600; 10 Graduate Institute of Chinese Medicine, China Medical University, Taichung 404; 11 Division of Cardiology, Department of Medicine, China Medical University Hospital, Taichung 404; 12 Department of Chinese Medicine, Wan Fang Hospital, Taipei Medical University, Taipei 116; 13 Graduate Institute of Pharmacognosy, College of Pharmacy, Taipei Medical University, Taipei 110; 14 Department of Biological Science and Technology, China Medical University, Taichung 404; 15 Department of Biotechnology, Asia University, Taichung 413, Taiwan, R.O.C

**Keywords:** deguelin, DNA damage, comet assay, DNA repair, human lung cancer NCI-H460 cells

## Abstract

It has been shown that deguelin, one of the compounds of rotenoids from flavonoid family, induced cytotoxic effects through induction of cell cycle arrest and apoptosis in many types of human cancer cell lines, but deguelin-affected DNA damage and repair gene expression (mRNA) are not clarified yet. We investigated the effects of deguelin on DNA damage and associated gene expression in human lung cancer NCI-H460 cells *in vitro*. DNA damage was assayed by using the comet assay and DNA gel electrophoresis and the results indicated that NCI-H460 cells treated with 0, 50, 250 and 500 nM deguelin led to a longer DNA migration smear based on the single cell electrophoresis and DNA fragmentation occurred based on the examination of DNA gel electrophoresis. DNA damage and repair gene expression (mRNA) were evaluated by using real-time PCR assay and the results indicated that 50 and 250 nM deguelin for a 24-h exposure in NCI-H460 cells, decreased the gene levels of breast cancer 1, early onset (*BRCA1*), DNA-dependent serine/threonine protein kinase (*DNA-PK*), *O*^6^-methylguanine-DNA methyltransferase (*MGMT*), p53, ataxia telangiectasia mutated (*ATM*) and ataxia-telangiectasia and Rad3-related (*ATR*) mRNA expressions. Collectively, the present study showed that deguelin caused DNA damage and inhibited DNA damage and repair gene expressions, which might be due to deguelin-inhibited cell growth *in vitro*.

## Introduction

Deguelin, one of the most critical rotenoids from the flavonoid family, derived from the natural plants in the *Mundulea sericea* family, has been shown to be effective as a chemopreventive and therapeutic agent against different cancer cells such as tumors of the colon, lung and breast (1–3). The functions of human cancer cell lines through the induction of cell cycle arrest and apoptosis can be down-regulated for specific cell survival proteins, including Akt and mitogen-activated protein kinase (MAPK) (4–6). Furthermore, deguelin inhibited the transcriptional regulation of ornithine decarboxylase (7), NF-κB gene expression (8,9) and hypoxia-inducible factor-1α (HIF-1α) (10).

DNA damage is associated with diseases such as neuro-degeneration in age-related disease, cerebral ischemia and brain trauma (11). Thus, agent-induced DNA damage may lead to cell mutation and then cause malignancy (12,13). To fully understand the actions of anticancer drugs is critical and can offer more information regarding the anticancer drug-induced side effects in patients.

Although substantial evidence has shown that deguelin induced cell death of human cancer cell lines, there is no information to address the effects of deguelin-provoked DNA damage in human lung cancer cells. The purpose of the present study was to investigate the effects of deguelin on DNA damage and DNA repair associated gene expression (mRNA) in human lung cancer NCI-H460 cells. Our results revealed that deguelin induced DNA damage and inhibited DNA associated gene expression in NCI-H460 cells *in vitro*.

## Materials and methods

### Chemicals and reagents

Deguelin, dimethyl sulfoxide (DMSO), propidium iodide (PI), Tris-HCl and Triton X-100 was obtained from Sigma-Aldrich Corp. (St. Louis, MO, USA). RPMI-1640 medium, fetal bovine serum (FBS), L-glutamine, penicillin-streptomycin and trypsin-EDTA were obtained from Gibco Life Technologies (Grand Island, NY, USA).

### Cell culture

The human lung cell line (NCI-H460) was purchased from the Food Industry Research and Development Institute (Hsinchu, Taiwan) and maintained at 37°C with 5% CO_2_ and 95% air in RPMI-1640 medium supplemented with 10% FBS, 2 mM L-glutamine, 100 units/ml penicillin and 100 μg/ml streptomycin. The medium was changed every 2 days (14). Deguelin was dissolved in DMSO and added directly to cell culture medium at a final concentration of 0.5% DMSO. This concentration had no effect on cell growth or other assays.

### PI exclusion method and flow cytometric assay

Approximately 2×10^5^ cells/well of NCI-H640 cells in 12-well plates were incubated with deguelin at final concentrations of 0 (vehicle, 0.5% DMSO), 50, 250 and 500 nM and for 24 h, or the cells were treated with 250 nM deguelin for 0, 24, 48 and 72 h. Cells from each treatment were stained with PI (5 μg/ml) and analyzed by flow cytometry (Becton-Dickinson, San Jose, CA, USA) and cell viability was calculated as previously described (15,16).

### Comet assay

NCI-H460 cells at a density of 2×10^5^ cells/well in 12-well plates were incubated with 0 (vehicle, 0.5% DMSO), 50, 250 and 500 nM degulein and 5 μM hydrogen peroxide (H_2_O_2_, positive control) for 48 h in RPMI-1640 medium grown at 37°C in 5% CO_2_ and 95% air. Cells were harvested for the examination of DNA damage using the comet assay as previously described (17,18). Comet tail length was calculated and quantified using the TriTek CometScore™ software image analysis system (TriTek Corp., Sumerduck, VA, USA) as previously described (18).

### DNA gel electrophoresis

NCI-H460 cells (1×10^6^ cells/well) seeded in 6-well plates were incubated with degulein at final concentrations of 0 (vehicle, 0.5% DMSO), 50, 250 and 500 nM for 48 h. Cells from each treatment were individually isolated by using DNA isolation kit (Genemark Technology Co., Ltd., Tainan, Taiwan) (19). The DNA electrophoresis was carried out in 1.5% agarose gel in Tris-borate EDTA (TBE) buffer (Amresco, Solon, OH, USA) at 15 V for 2 h. DNA was stained with ethidium bromide (EtBr, Sigma-Aldrich Corp.), then examined and photographed by fluorescence microscope as previously described (20,21).

### Real-time PCR analysis

Approximately 1×10^6^ cells/well of NCI-H460 cells in 6-well plates were incubated with or without 0, 50 and 250 nM degulein for a 24-h treatment in RPMI-1640 medium grown at 37°C in 5% CO_2_ and 95% air. The total RNA from each treatment was extracted by using the Qiagen RNeasy Mini Kit (Qiagen, Inc., Valencia, CA, USA) as previously described (14,22). Briefly, RNA samples were reverse-transcribed for 30 min at 42°C with High Capacity cDNA Reverse Transcription Kit according to the standard protocol of the supplier (Applied Biosystems, Carlsbad, CA, USA). For quantitative PCR from each sample that was performed in the conditions: 2 min at 50°C, 10 min at 95°C, and 40 cycles of 15 sec at 95°C, 1 min at 60°C using 1 μl of the cDNA reverse-transcribed as described above, 2X SYBR-Green PCR Master Mix (Applied Biosystems) and 200 nM of forward and reverse primers as shown in Table I. Finally, each assay was run on an Applied Biosystems 7300 real-time PCR system in triplicates and expression fold-change was derived using the comparative C_T_ method (15,18).

### Statistical analysis

The data are presented as the mean ± SD and Student’s *t*-test was used to analyze differences between deguelin-treated and untreated (control) groups. All the statistical analyses were performed, and p<0.05 was considered statistically significant.

## Results

### Flow cytometric assay for the effects of deguelin on the percentage of viable NCI-H460 cells

**Figure 1:**
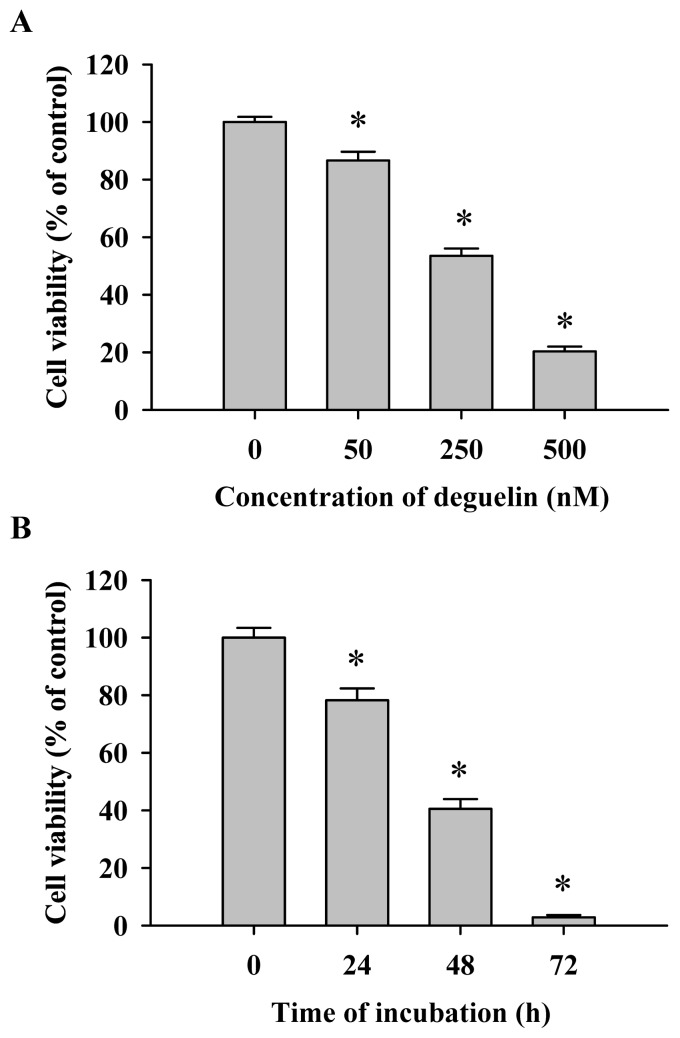
Deguelin decreased the percentage of viable human lung cancer NCI-H460 cells. Cells at a density of 2×10^5^ cells/well were placed in 12-well plates and incubated with deguelin at final concentrations of 0 (vehicle, 0.5% DMSO), 50, 250 and 500 nM for 48 h (A), or cells were treated with 250 nM deguelin for 0, 24, 48 and 72 h (B). Cells from each treatment were stained with PI (5 μg/ml) and analyzed by flow cytometry as described in Materials and methods. ^*^p<0.05 was considered significant when compared with vehicle control cells (0 μM).

Cells were treated with various concentrations (0, 50, 250 and 500 nM) of deguelin for 48 h or were treated with 250 nM of deguelin for 0, 24, 48 and 72 h. The cells from each treatment were collected for the measurement of percentage of viable NCI-H460 cells. The results shown in Fig. 1 indicate that deguelin decreased the cell viability and these effects are dose- and time-dependent (Fig. 1).

### Comet assay for the effects of deguelin-triggered DNA damage in NCI-H460 cells

**Figure 2:**
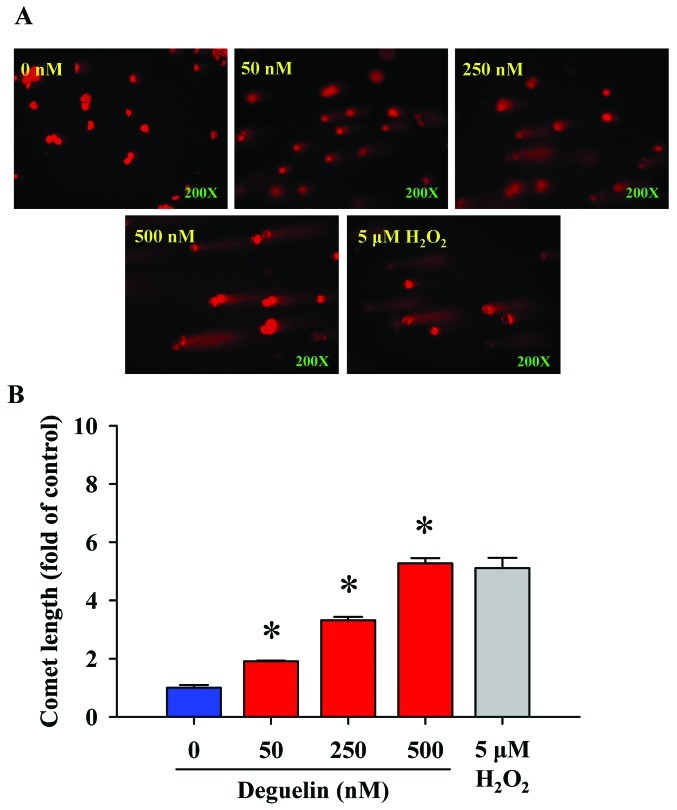
Deguelin induced DNA damage in NCI-H460 cells as determined by comet assay. Cells at a density of 2×10^5^ cells/well in 12-well plates were exposed to deguelin at final concentrations of 0, 50, 250 and 500 nM, and 5 μM H_2_O_2_ (positive control) for 48 h and DNA damage was determined by comet assay as described in Materials and methods. (A) Representative picture of comet assay for dose-dependent effects; (B) comet length (fold of control) was quantified using the TriTek CometScore software image analysis system. ^*^p<0.05 was considered significant when compared with vehicle control cells (0 μM).

We investigated that deguelin-induced DNA damage of NCI-H460 cells *in vitro*. The comet assay was selected for determining DNA damage and the results are shown in Fig. 2, indicating that deguelin provoked DNA damage in NCI-H460 cells in a dose-dependent manner. The higher concentration of deguelin led to a longer DNA migration smear (comet tail). It is well documented that H_2_O_2_ is a highly reactive oxygen species and it has been used as positive control for numerous studies (23,24). The results from present studies indicated that 5 μM H_2_O_2_-induced comet tail occurred and was used as a positive control.

### DNA gel electrophoresis for the effects of deguelin-induced DNA damage and fragmentation in NCI-H460 cells

**Figure 3:**
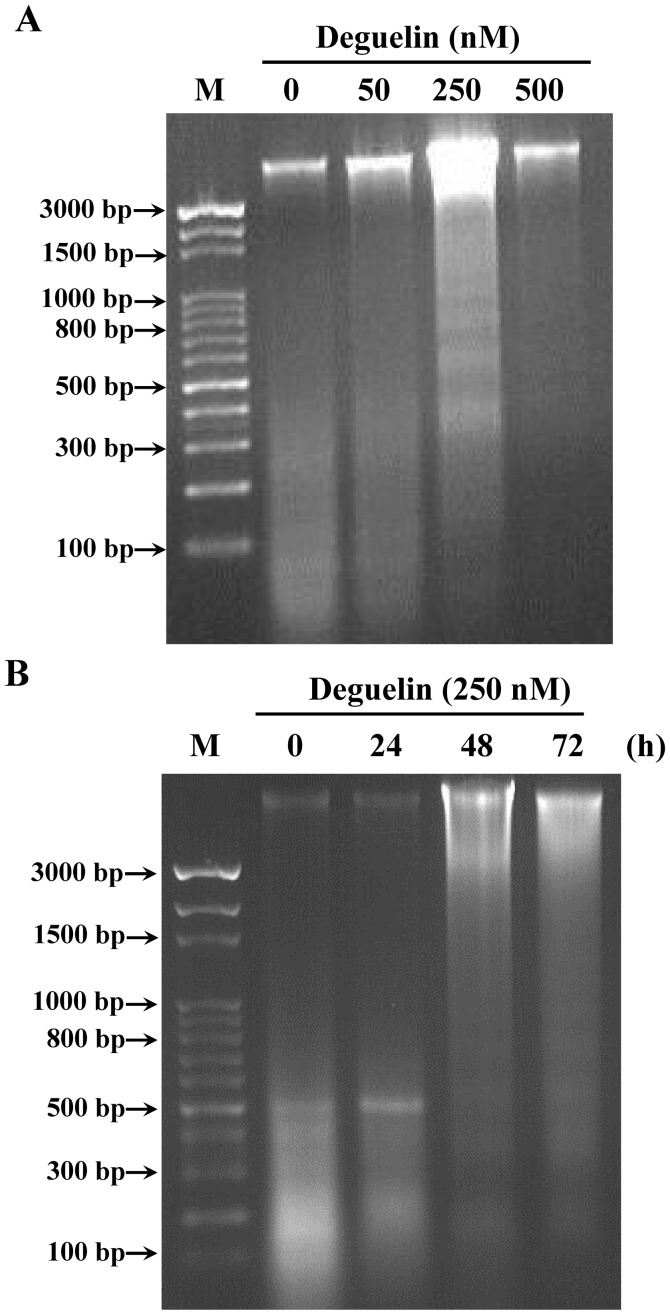
Deguelin-induced DNA damage in NCI-H460 cells was determined by agrose DNA gel electrophoresis. Cells at a density of 1×10^6^ cells/well were placed in 6-well plates were incubated with deguelin at final concentrations of 0, 50, 250 and 500 nM for 48 h. Cells were collected and DNA were isolated from each treatment for gel electrophoresis described in Materials and methods. M, marker.

In comet assay, we found that deguelin induced DNA damage in NCI-H460 cells. Thus, DNA gel electrophoresis was used to investigate whether or not deguelin causes DNA fragmentation in NCI-H460 cells. Thus, DNA was isolated from NCI-H460 cells after treatment with deguelin for 48 h and then DNA fragments were determined by DNA gel electrophoresis. The results showed that deguelin induced DNA damage and fragments in NCBI-H460 cells in a dose- and time-dependent manner (Fig. 3). The highest dose of deguelin (500 nM) incubation of NCI-H460 cells led to more DNA damage and fragments than that of low dose (50 nM) deguelin incubation.

### Real-time PCR for examining the effects of deguelin on DNA damage and repair gene expression in NCI-H460 cells

**Figure 4:**
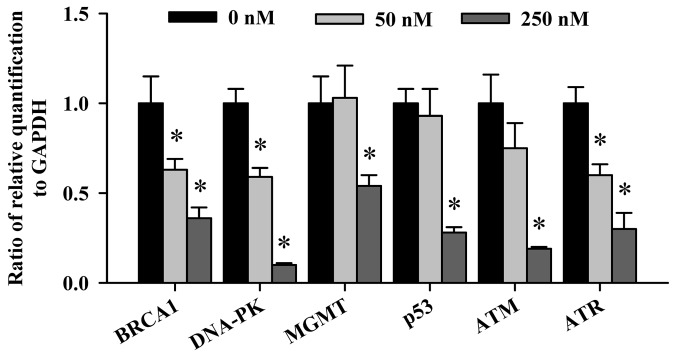
Deguelin-inhibited DNA damage and repair gene expression in NCI-H640 cells were determined by real-time PCR. Total RNA was extracted from the NCI-H640 cells after treatment with 0, 50 and 250 nM deguelin for 24 h, and RNA samples were reverse-transcribed for real-time PCR as described in Materials and methods. The ratios of BRCA1, DNA-PK, MGMT, p53, ATM and ATR mRNA/GAPDH are shown and data represent the mean ± SD of three experiments. ^*^p<0.05 was considered significant.

**Figure 5:**
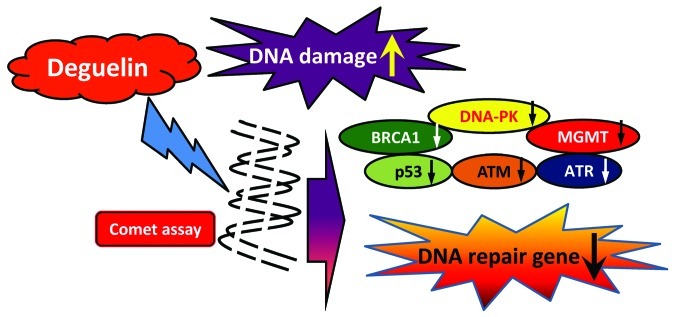
The possible flow chart for deguelin-inhibited gene expression of DNA damage and repair in human lung cancer NCI-H460 cells.

Based on the above results, deguelin induced DNA damage and fragments in NCI-H460 cells. We further investigated the effects of deguelin on gene expression of DNA damage and repair in NCI-H460 cells. We also used DNA agarose gel electrophoresis for examining the products (Fig. 3). The real-time PCR results are shown in Fig. 5 and indicate that all the examined gene expressions associated with DNA damage and repair such as the *BRCA1*, *DNA-PK*, *MGMT*, *p53*, *ATM* and *ATR* mRNA were decreased (Fig. 4) in NCI-H460 cells after a 24-h treatment of deguelin. Especially, the gene levels of *BRCA1*, *DNA-PK*, *ATM* and *ATR* expression were inhibited dose-dependently in NCI-H460 cells. However, the gene levels of *MGMT* and *p53* mRNA expression were decreased in NCI-H460 cells only at high dose of deguelin exposure.

## Discussion

Several reports have demonstrated that deguelin can induce cytotoxic effects and induce apoptosis in many human cancer cell lines (1,4,26–28). However, there is no report addressing deguelin-induced DNA damage in human lung cancer cells. In the present study, a dose-dependent increase in DNA damage (Fig. 2) was observed in human lung cancer NCI-H460 cells associated with a loss of cell viability in a dose- and time-dependent manner (Fig. 1). These findings indicated: i) DNA damage from comet assay (single cell gel electrophoresis) occured in the tail moment of the comets from NCI-H460 cells, the longer the comet tail the higher the DNA damage (Fig. 2) in a dose-dependent manner; ii) DNA fragments from DNA gel electrophoresis indicated that high dose of deguelin treatment led to high fragmentation in NCI-H460 cells (Fig. 3).

Comet assay is a highly sensitive technique for DNA damage examination and thus it has been used for screening the effects of agent on DNA damage in cells (28–30). Furthermore, a measurement for trend-break formation during the process of excision repair of DNA could be used (31,32). In our earlier studies, we have shown that deguelin induced apoptosis in human cancer cell lines (data not shown), but we also found that deguelin induced apoptosis based on DNA fragmentation occur in NCI-H460 cells after exposure to deguelin from DNA agarose gel electrophoresis assay (Fig. 3). Our earlier studies also showed that deguelin-induced apoptosis may be through the production of reactive oxygen species (ROS) in NCI-H460 cells (data not shown); thus, we suggest that deguelin induced DNA damage may be via the production of ROS. Further studies are needed to establish the role of the interaction of deguelin with DNA in cancer cells.

Numerous evidence has shown that in cells, agents can induce DNA damage which can be reduced by DNA repair system through eliminating DNA lesions (33–35). In the present study, our results from the comet assay (Fig. 2) and DNA gel electrophoresis indicated that deguelin-induced DNA damage (Fig. 3) in NCI-H460 cells. Furthermore, results were obtained from real-time PCR (Fig. 4) which indicated that DNA repair gene expression including *BRCA1*, *DNA-PK*, *MGMT*, *p53*, *ATM* and *ATR* were inhibited in deguelin-treated NCI-H460 cells. Importantly, the gene levels of *BRCA1*, *DNA-PK*, *ATM*, *ATR* and *DNA-PK* expressions were reduced dose-dependently.

Cells after stimulation by agents cause DNA damage and the DNA damage checkpoints are signal transduction pathways which are involved in the cell cycle and cellular responses to DNA damage in order to maintain genomic integrity (36–38). Especially, the ATM and ATR are two master checkpoint kinases activated by double-stranded DNA breaks (DSBs) (39). In UV-damaged DNA and incompletely replicated DNA, the ATR kinase is responsible for initiating the DNA damage checkpoint (40). BRCA1 (tumor suppressor) plays critical roles in DNA repair, cell cycle checkpoint control and maintenance of genomic stability in human breast and ovarian cancer (41). Moreover, DNA-PK plays a critical role in DNA damage repair (42). MGMT reduces cytotoxicity of therapeutic or environmental alkylating agents (43).

In conclusion, the possible flow charts for deguelin-affected DNA damage in human lung cancer NCI-H460 cells are summarized in Fig. 5 which indicates that deguelin induced DNA damage followed by the inhibition of DNA repair associated gene expressions (mRNA) including BRCA1, DNA-PK, MGMT, p53, ATM and ATR, resulting in maintenance of DNA damage (Fig. 5).

## Figures and Tables

**Table I:** Primer sequences used for real-time PCR.

Primer name	Primer sequence
BRCA1	F: CCAGGGAGTTGGTCTGAGTGA R: ACTTCCGTAAGGCATCGTAACAC
DNA-PK	F: CCAGCTCTCACGCTCTGATATG R: CAAACGCATGCCCAAAGTC
MGMT	F: CCTGGCTGAATGCCTATTTCC R: TGTCTGGTGAACGACTCTTGCT
p53	F: GGGTTAGTTTACAATCAGCCACATT R: GGGCCTTGAAGTTAGAGAAAATTCA
ATM	F: TTTACCTAACTGTGAGCTGTCTCCAT R: ACTTCCGTAAGGCATCGTAACAC
ATR	F: GGGAATCACGACTCGCTGAA R: CTAGTAGCATAGCTCGACCATGGA
GAPDH	F: ACACCCACTCCTCCACCTTT R: TAGCCAAATTCGTTGTCATACC

The human DNA sequences were evaluated using the Primer Express software and each assay was run on an Applied Biosystems 7300 real-time PCR system. Each assay was conducted at least trice to ensure reproducibility. BRCA1, breast cancer gene 1; DNA-PK, DNA-dependent serine/threonine protein kinase; MGMT, *O*^6^-methylguanine-DNA methyltransferase; ATM, ataxia telangiectasia mutated; ATR, ataxia-telangiectasia and Rad3-related; GAPDH, glyceraldehydes-3-phosphate dehydrogenase.
